# Voided volume may not impact stone outcomes: Review of a large institutional nephrolithiasis cohort

**DOI:** 10.1002/bco2.216

**Published:** 2023-04-06

**Authors:** Kevin Shee, Carter Chan, Heiko Yang, Wilson Sui, Max Bowman, Fadl Hamouche, Leslie Bernal Charondo, Sunita Ho, Thomas Chi, Marshall L. Stoller

**Affiliations:** ^1^ Department of Urology UCSF San Francisco California USA

**Keywords:** 24‐h urine, nephrolithiasis, outcomes, registry, voided volume

## Abstract

**Background:**

Urologic guidelines universally recommend increasing fluid intake for kidney stone prevention. Increased voided volume is thought to help reduce stone recurrence and severity, but supporting evidence is limited.

**Patients and Methods:**

Nephrolithiasis outcomes and 24‐h urine data for patients from the Registry for Stones of the Kidney and Ureter (ReSKU), a registry of nephrolithiasis patients collected between 2015 and 2020, were retrospectively analysed. Outcome was stone events, either an office visit where a patient reports symptomatic passage of stones or surgery for stone removal.

**Results:**

We identified 450 stone patients with 24‐h urine and kidney stone outcome data. There was no significant difference in 24‐h voided volume between patients with one stone event and patients with two or more stone events. On multivariable logistic regression, after controlling for age, gender, BMI, and 24‐h sodium and creatinine per kilogram, no significant associations were found between voided volume and stone events. There was a statistically significant negative correlation noted between voided volume and stone events in calcium oxalate dihydrate stone formers (Spearman *R* = −0.42, *p* = 0.04), but not others.

**Conclusions:**

Twenty‐four‐hour voided volume was not associated with stone events in a large institutional cohort, and subset analysis reveals that some stone formers may benefit more from increased voided volume than others; identifying such patients represents a novel precision medicine opportunity.

## 1 INTRODUCTION

Kidney stone disease affects nearly one in 11 people in the United States, and it is a disease associated with a great degree of morbidity and high rate of health care utilization.^1^ Increasing fluid intake with the goal of increasing urinary volume has long been a key tenet in the prevention of stone disease. The 2019 AUA kidney stone guidelines recommend titrating fluid intake to achieve a urine volume of at least 2.5 L daily.^2^ The 2021 EAU stone prevention guidelines similarly recommend achieving a urinary volume of 2 to 2.5 L daily.^3^

The most cited study supporting these guidelines is the prospective randomized clinical trial by Borghi et al. in 1996 showing that patients started on a high fluid intake program with a target urine volume of more than 2 L daily had 50% lower stone recurrence rates and longer time to first stone recurrence compared with controls.^4^ Although additional studies supporting higher fluid intake for nephrolithiasis prevention have been reported,^5^, ^6^, ^7^, ^8^, ^9^ they are limited as pointed out by a recent Cochrane review.^10^ The authors revealed that the Borghi study was the only study that met their strict inclusion criteria. They further determined that the Borghi study was at risk of multiple biases, including selection bias, performance bias, detection bias, and reporting bias.

By identifying patients from a novel UCSF database, the Registry for Stones of the Kidney and Ureter (ReSKU),^11^ with outcomes data and 24‐h urine data, we sought to more rigorously examine the association between initial 24‐h voided volume and reported stone events.

## 2 PATIENTS AND METHODS

### 2.1 Study participants

After 2015, patients presenting to the University of California San Francisco (UCSF) Urology clinic for stone disease management were enrolled into the Registry for Stones of the Kidney and Ureter (ReSKU) database, a prospective, high‐quality, automated nephrolithiasis registry that extracts data directly from the electronic health record (EHR). The enrolment protocol and other details have been fully described previously.^11^ Nephrolithiasis outcome data was available for patients from 2015 to 2020. Patients enrolled in the ReSKU database with at least one prospectively collected 24‐h urine test (Litholink 24‐h urine test; Labcorp) were included. Surgeries included percutaneous nephrolithotomy (PCNL), ureteroscopy (URS), extracorporeal shock wave lithotripsy (ESWL), cystolitholapaxy, open stone removal, and lap/robotic stone removal. Patients with cystine stones were excluded from analysis. All enrolled patients provided informed consent, and the study was approved by the University of California San Francisco institutional review board and Human Research Protection Program (Protocol 14‐14533). All research was performed in accordance with the Declaration of Helsinki.

### 2.2 Outcomes and study variables

Demographic data on patient age, race, gender, and BMI were analysed; 24‐h urine data for voided volume, 24‐h urine sodium (Na24), a surrogate for dietary sodium intake, and creatinine per kilogram (CrKg24), a surrogate for kidney function, were obtained. The primary nephrolithiasis outcome was number of stone events, defined as a symptomatic passage of stones or a surgery for stone removal. To prevent overcounting due to incomplete clearance of stones due to technical or surgical factors, patients who had a symptomatic stone episode without passage of stones and a stone removal surgery within 90 days of that episode were only counted as a singular stone event.

### 2.3 Statistical analysis

Nonparametric Mann–Whitney and Kruskal–Wallis analysis of variance tests were used to compare continuous variables between two or three groups, respectively. Chi‐squared tests were used to compare categorical variables between two groups. Multivariable logistic regression analysis was performed to determine the impact of voided volume on stone events while controlling for gender, age, BMI, CrKg24, and Na24. Spearman correlations were performed to determine the impact of voided volume on stone events for each primary stone composition. Statistical analyses were performed using R 3.4.0 (R Foundation, Vienna, Austria) and validated with GraphPad Prism v7.

## 3 RESULTS

The number of patients with both outcomes and 24‐h urine data in the ReSKU dataset included 450 patients from 2015 to 2020. Demographic and clinical characteristics of the cohort are summarized in Table 1. Of these, 233 patients (51.8%) were men, and 293 patients (63%) were White/Caucasian. Median BMI in the cohort was 25.8 kg/cm^2^ (IQR 7.3). Median CrKg24 was 19.8 mg/kg/24 h (IQR 7.3). Median 24‐h voided volume was 2.1 L (IQR 1.2); 196 patients (43.6%) had one stone event, and 254 (56.4%) had two or more stone events.

**TABLE 1:** Demographic and clinical characteristics of the nephrolithiasis patients with 24‐h urine data

	Cohort
	*N* = 450
Age (median, IQR), years	55 (23)
Gender (*n*, % Male)	233 (51.8)
Ethnicity (*n*, %)	
Caucasian	293 (63.6)
Asian	55 (12.2)
Hispanic/Latino	42 (9.1)
Black	14 (3.0)
Other	46 (10.2)
BMI (median, IQR), kg/cm^2^	25.8 (7.3)
CrKg24 (median, IQR), mg/kg/24 h	19.8 (7.3)
Na24 (median, IQR), mmol/24 h	146.9 (91.9)
Vol24 (median, IQR), litres/24 h	2.1 (1.2)
Stone events (*n*, %)	
1	196 (43.6)
2 or more	254 (56.4)

Abbreviations: BMI, body mass index; Cr24Kg, 24‐h creatinine per kilogram; IQR, interquartile range; Vol24, 24‐h voided volume.

**FIGURE 1:**
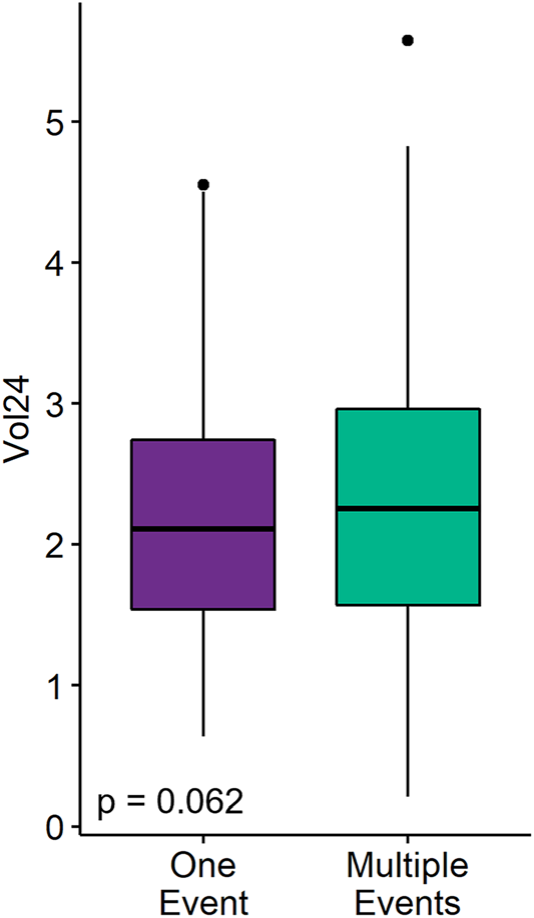
Voided volume not significantly different between patients with single stone events and patients with multiple stone events

**FIGURE 2:**
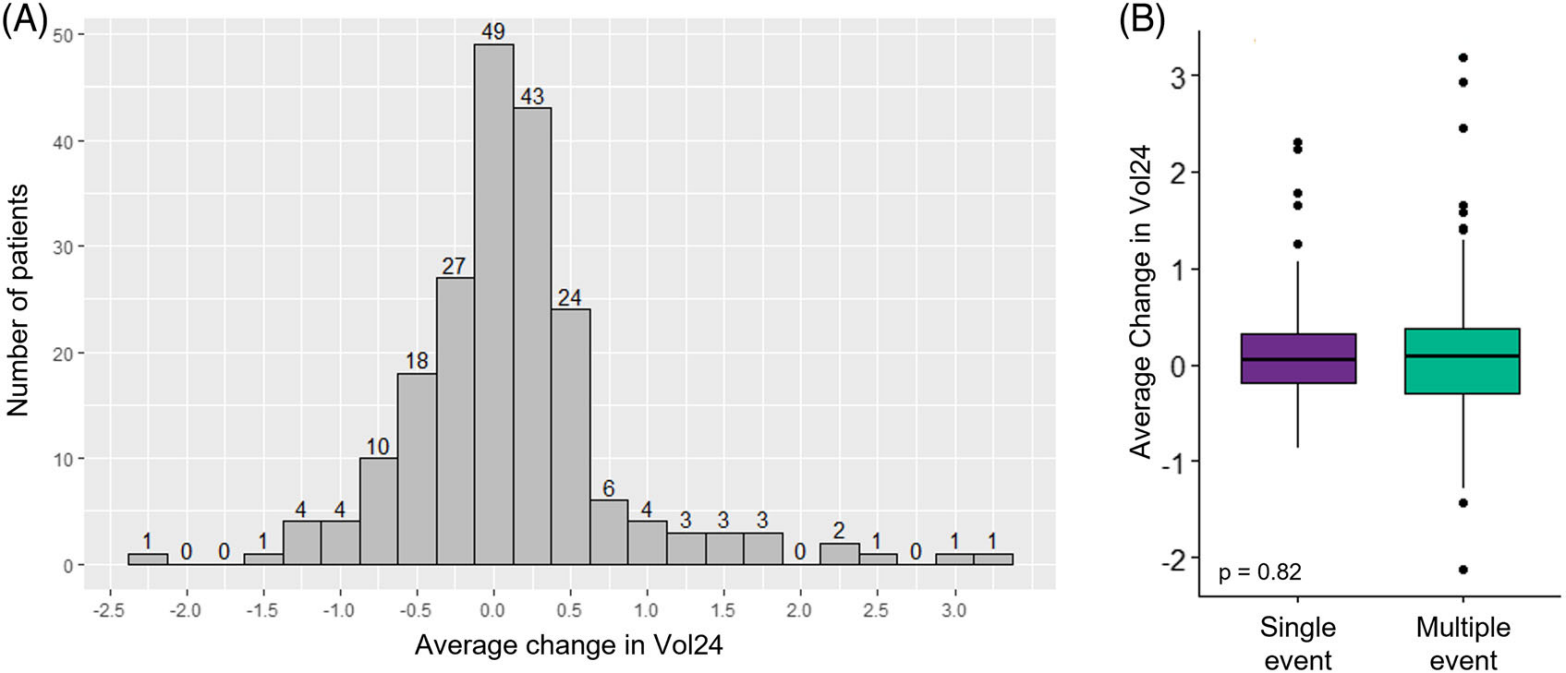
Change in voided volume not significantly different between patients with single stone events and patients with multiple stone events

There was no significant difference in 24‐h voided volume between patients with one stone event and patients with two or more stone events (Kruskal–Wallis *p* = 0.06; Figure 1). Multivariable logistic regression analysis demonstrated that female gender (OR 1.93, 95% CI 1.25–3.00, *p* = 0.0031) and higher BMI (OR 1.04, 95% CI 1.00–1.08, *p* = 0.03), but not voided volume (OR 1.04, 95% CI 0.83–1.33, *p* = 0.68) were associated with multiple stone events (Table 2). Na24 and Cr24Kg were found to be highly correlated with voided volume (*R* = 0.31 and 0.22, respectively, *p* < 0.0001 for both). Additionally, we identified 205 patients within our cohort who have had at least two 24‐h urine tests. We found that the average change in voided volume between the two urine collections was minimal (Figure 2A). Furthermore, we found no significant difference in the average change in voided volume between patients with one stone event and patients with two or more stone events (Kruskal–Wallis *p* = 0.82; Figure 2B).

**TABLE 2:** Multivariable logistic regression models for having more than one stone event

Characteristic	More than one stone event		
	*p* value	OR	95% CI
Gender (F)	0.0031	1.93	(1.25, 3.00)
Age	0.79	1.0	(0.98, 1.01)
BMI	0.041	1.04	(1.00, 1.08)
Cr24Kg	0.64	0.98	(0.94,1.03)
Na24	0.68	1.0	(1.0, 1.0)
Vol24	0.68	1.04	(0.83,1.33)

Abbreviations: BMI, body mass index; CI, confidence interval; Cr24Kg, 24‐h creatinine per kilogram; F, female; Na24, 24‐h sodium; OR, odds ratio; Vol24, 24‐h voided volume.

**FIGURE 3:**
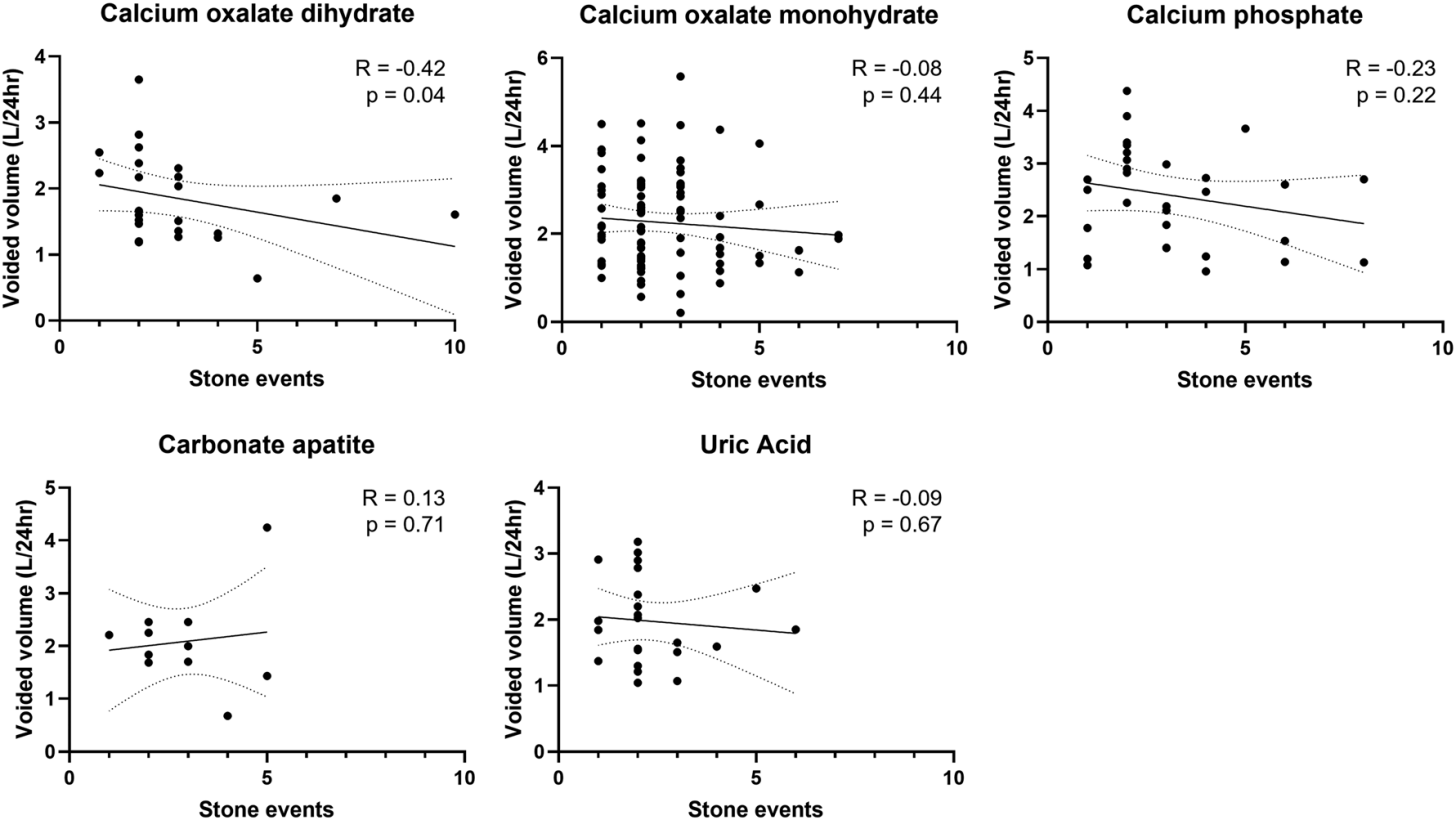
Correlative analysis between voided volume and stone events, divided by stone type

The stone cohort was then divided based on primary stone composition, and correlation analysis was performed to determine whether voided volume may be more predictive of stone events in patients with specific stone compositions. There was a statistically significant negative correlation noted between voided volume and stone events in calcium oxalate dihydrate stone formers (Spearman *R* = −0.42, *p* = 0.04; Table 3; Figure 3). Additional significant relationships between voided volume and stone events among other types of stone formers were not observed.

**TABLE 3:** Correlative analysis between voided volume and stone events, divided by stone type

Correlation with voided volume (Spearman *R*)	Stone events	
Primary stone type	*R*	*p* value
Calcium oxalate dihydrate (*n* = 24)	−0.42	0.04
Calcium oxalate monohydrate (*n* = 98)	−0.08	0.44
Calcium phosphate apatite (*n* = 29)	−0.23	0.22
Carbonate apatite (*n* = 11)	0.13	0.71
Uric acid (*n* = 23)	−0.09	0.67

## 4 DISCUSSION

A key recommendation by urologic guidelines for the medical management of stone disease is to increase voided volume to at least 2 L per day, which is supported by multiple early studies. One of the first clinical studies, Frank et al. in 1966 compared the incidence of urinary stone formation from two desert towns in Israel, demonstrating that education about increased fluid intake significantly increased urine volume and decreased the incidence of urolithiasis during a 3‐year follow‐up period.^12^ Similarly, multiple studies have linked increased incidence and prevalence of kidney stones to dehydration, for example in regions of the country with drier climates^13^ or in long‐distance runners.^14^ Another early study, Strauss et al. in 1982 demonstrated that recurrent stone formers with a new episode of urolithiasis during a 2‐year evaluation period had increased urine volume to a lower extent compared with patients who were stone free.^15^ However, each of these studies had significant limitations and were subject to multiple biases that limit their validity; for example, in the Frank et al. study, the town that received education was directly interviewed by the study team while the town without education was evaluated based on records of local physicians.

A recent Cochrane review investigating the role of increased water intake for the prevention of kidney stones identified only a single randomized clinical trial that met criteria for quantitative analysis.^10^ In this randomized controlled study by Borghi et al., 199 participants were randomized after their first calcium stone event to either an increased water intake program, aiming for urine volume of greater than 2 L per day, or not. After a follow‐up period of 5 years, the increased water intake group demonstrated 50% lower stone recurrence rates and longer time to first recurrence compared with controls.^4^ Although eligible for inclusion in the Cochrane review, the authors noted that the study was limited by small sample size, insufficient information about the randomization and blinding processes, and overall low stone recurrence rate over the follow‐up period.

Using a well‐described registry of urolithiasis patient outcomes and 24‐h urine data, we sought to determine whether voided volume was associated with kidney stone recurrence. No significant differences were found between the 24‐h voided volume of patients with single stone events or multiple stone events by initial non‐parametric testing, confirmed by multivariable regression modelling which controls for differences in known risk factors of gender, age, BMI, and kidney function in our multivariable regression models. Overall, there was little change in voided volume in patients who had multiple 24‐h urine tests, and change in voided volume was not significantly different between patients with single stone events and patients with multiple stone events. Furthermore, correlation analyses based on patient primary stone composition revealed a statistically significant association between voided volume and stone events in calcium oxalate dihydrate stone formers, but not others. These findings, combined with a lack of substantial high‐quality clinical trials on the subject, call into question the undisputed recommendation to increase voided volume in all patients with urolithiasis. Instead, a precision medicine approach that involves identification of which patients may benefit most from increased voided volume represents an important future avenue of research.

We also found in our multivariable regression analyses that BMI is associated with stone recurrence and stone events, which is a well‐described risk factor in literature.^16^ Female gender was also found to be associated with disease severity; although nephrolithiasis is traditionally thought to affect males more than females, recent trends demonstrate increasing proportions of female nephrolithiasis over the past few decades.^17^, ^18^ Together, these results suggest that both younger patients and female patients in our study cohort may have more severe disease compared with that of the general population. While this may reflect a shifting demographic trend for nephrolithiasis, this also may reflect the nature of the study centre as a tertiary stone clinic, which attracts a greater proportion of patients with relatively complex stone disease compared with other populations. Finally, we found that voided volume was highly correlated with 24‐h urine sodium, which is consistent with prior work demonstrating that increased dietary sodium leads to significantly increased urinary voided volume with no statistically significant change in urinary calcium, oxalate or uric acid.^19^ In clinical practice, stone management guidelines recommend that patients limit their sodium intake to improve outcomes,^2^ which may inadvertently lead to decrease fluid intake and voided volume. Together, this suggests a nuanced balance between recommending decreased sodium intake and increased voided volumes, which warrants further study.

We would be remiss to not mention the potential impact of the Internet and social media to the findings of our study. In the digital age, the Internet has provided access to a wealth of information for both patients and providers about the diagnosis and management of disease. Furthermore, there is a growing impact of social media platforms such as Twitter, where prominent Urologists can disseminate and share information to the public. Specifically for nephrolithiasis, Huang et al. in 2021 show doubling of online searches for videos about kidney stones between 2015 and 2019.^20^ Another systematic review showed that social media and online search engine information regarding dietary and fluid management aspects of stone disease was valuable and accurate.^21^ Although not directly addressed by our study, it is possible that early patient access to online information encouraging increased voided volume may have contributed to the lack of association between stone events and voided volume.

A major limitation is the single‐centre nature of the study, which may introduce selection bias and limit the generalizability of the results to other populations. Additionally, there are limitations due to the nonrandomized and retrospective nature of the study, such as confounding and lack of standardized follow‐up and treatment. Another potential bias is due to the nature of the 24‐h urine test, where patients may purposely increase their fluid intake during collection in a manner that is unrepresentative of typical behaviour. The study is further limited by potential recall bias as some outcome data, such as number of childhood stone events, is based on patient memory, and patients with longstanding history of nephrolithiasis may be biased towards recalling more stone events. However, prior studies have shown that stone patients tend to have accurate recollection of stone episodes when compared with medical records.^22^ Finally, only 41% of the 450 patients had stone analysis data, further limiting power of analysing the data by primary stone type. Planned future research includes incorporation of additional patients beyond 2020 and the addition of improved outcome measures such as time to recurrence and objective assessments of stone recurrence such as CT imaging.

Despite these limitations, the findings provide evidence challenging conventional dogma that increased voided volume leads to decreased stone recurrence and severity. Additional prospective randomized clinical trials and precision medicine studies may further shed light on which patients may benefit from increased voided volume.

## 5 CONCLUSION

In this study, we show that 24‐h voided volume is not significantly associated with stone outcomes in 450 nephrolithiasis patients. Subset analysis revealed a moderate negative correlation between voided volume and stone outcomes in calcium oxalate dihydrate stone formers, but not others. These findings suggest that established guideline recommendations of increasing voided volume in all nephrolithiasis patients may be more nuanced than initially thought and that identifying populations that may benefit from increased voided volume represents a logical next step.

## CONFLICT OF INTEREST

All authors declare that they have no conflict of interest regarding the material presented in this manuscript. All authors declare that they approve submission of the final article.

## AUTHOR CONTRIBUTIONS

KS, CC, SH, TC, and MS contributed to study conception and design, data collection and analysis, and manuscript writing. HY, WS, MB, FH, and LBC contributed to study data collection and analysis, and manuscript writing. All authors discussed the results and contributed to the final manuscript.

## Data Availability

Deidentified data that support the findings of this study are available on request from the corresponding author, MS. The data are not publicly available due to their containing information that could compromise the privacy of patients.
